# Giant Mixed-Material Gastric Bezoar With a Synchronous Jejunal Bezoar in a Young Woman With Pica and Autism Spectrum Disorder: A Case Report

**DOI:** 10.7759/cureus.116358

**Published:** 2026-09-16

**Authors:** Sergio Miguel Suárez Fuentes, Mauro A Cardenas, Valeria Gomez Marquez, Luis F Hernández González, José J Cervantes De Anda, Martín Rodríguez-Garza, Soledad M Castro Olmedo

**Affiliations:** 1 General Surgery, Hospital Regional “Valentín Gómez Farías”, Zapopan, MEX; 2 Surgery, “Presidente Lázaro Cárdenas del Río”, Instituto de Seguridad y Servicios Sociales de los Trabajadores del Estado (ISSSTE), Chihuahua, MEX; 3 General Surgery, Hospital Regional de Monterrey, Instituto de Seguridad y Servicios Sociales de los Trabajadores del Estado (ISSSTE), Monterrey, MEX

**Keywords:** autism spectrum disorder, bezoar, foreign body ingestion, gastric bezoar, intestinal obstruction, jejunal bezoar, laparotomy, metal bezoar, pica

## Abstract

Bezoars are conglomerates of indigestible material retained within the gastrointestinal tract and may produce obstruction, ulceration, bleeding, or perforation. Mixed-material bezoars containing nonfood foreign bodies are uncommon and may be particularly challenging in patients with persistent pica.

We report a 21-year-old woman with autism spectrum disorder (ASD), mild intellectual disability, previously diagnosed pica, and a seizure disorder treated with carbamazepine who presented after nearly one month of recurrent fever, colicky abdominal pain, progressive distension, early satiety, constipation, and nonbilious alimentary vomiting. Examination demonstrated abdominal distension and a firm, poorly defined epigastric-mesogastric mass without peritoneal irritation. Leukocytosis with neutrophilia was present. Abdominal radiography showed marked gastromegaly with mottled intragastric radiolucency, and computed tomography (CT) demonstrated a stomach almost completely occupied by heterogeneous solid material. Upper endoscopy confirmed a large gastric foreign-body mass. Exploratory laparotomy revealed marked gastromegaly and, unexpectedly, a separate bezoar obstructing the proximal jejunum that had not been identified preoperatively. A 15-cm anterior gastrotomy permitted removal of 3,000 g of material, and a 5-cm antimesenteric jejunotomy permitted removal of an additional 500 g. The two masses were not physically connected and contained plastic bags and bottle fragments, plastic caps, candy wrappers, adhesive tape, hair, plant fibers, and metallic staples. Both enteric incisions were closed in two layers, with an outer Lembert layer. Oral liquids were initiated on postoperative day 5 and advanced as tolerated; the patient was discharged on postoperative day 8 without leak, infection, or other surgical complications. Follow-up at one week after discharge and at one month showed favorable recovery.

This case highlights the diagnostic importance of considering synchronous distal bezoars in patients with severe pica and a massive gastric bezoar, particularly when preoperative imaging identifies only the dominant gastric lesion.

## Introduction

Bezoars are intraluminal conglomerates of material that resist normal digestion and gastrointestinal transit. Although uncommon, they remain clinically relevant because their presentation ranges from nonspecific dyspeptic symptoms to gastric outlet obstruction, small-bowel obstruction, pressure ulceration, bleeding, perforation, pancreatobiliary complications, and the need for urgent surgery [1]. Their composition is heterogeneous. Classical categories include phytobezoars, trichobezoars, pharmacobezoars, and lactobezoars, but contemporary reports increasingly describe xylobezoars and bezoars composed predominantly of plastics, metals, or mixed nonfood materials, particularly in the setting of pica [1].

Pica is the persistent ingestion of nonnutritive, nonfood substances and may occur in association with neurodevelopmental and psychiatric disorders. In a large pediatric study, pica was substantially more frequent among children with autism spectrum disorder (ASD), particularly when intellectual disability was also present, than among population controls [2]. Neurologic and psychiatric literature likewise emphasizes that pica can result in substantial gastrointestinal morbidity and that management requires attention not only to the mechanical complication but also to the underlying behavioral drivers [3]. The spectrum of ingested material can be broad; recent reports include plastic bezoars causing intestinal obstruction, paper-based xylobezoars, and mixed foreign-body bezoars in patients with ASD or pica [4-7].

The case presented here is unusual for several reasons. The total measured bezoar burden was 3.5 kg; the material was highly heterogeneous, including plastic, metallic, organic, and hair components; and two anatomically separate masses were present: one massive gastric bezoar and one synchronous proximal jejunal bezoar. Importantly, the jejunal lesion was not identified on preoperative computed tomography (CT) and was discovered only during laparotomy. Because there was no physical continuity between the gastric and jejunal masses, the presentation differs from classic Rapunzel syndrome, in which a trichobezoar extends continuously from the stomach into the small bowel. The case therefore illustrates both the consequences of severe pica and a practical diagnostic problem: a dominant gastric bezoar does not exclude a separate distal bezoar, even when cross-sectional imaging appears to show only one lesion.

## Case presentation

A 21-year-old woman was evaluated for an approximately one-month history of recurrent fever accompanied by progressively symptomatic gastrointestinal complaints. Her family described temperatures above 38°C during this period. She had colicky abdominal pain, progressive abdominal distension, early satiety, diaphoresis, constipation with a mild reduction in bowel movements, and recurrent nonbilious vomiting of alimentary/gastric contents. The prolonged course and increasing intolerance of oral intake prompted hospital evaluation.

Her relevant medical history included ASD diagnosed in childhood, mild intellectual disability, and previously diagnosed pica. She was verbal and able to participate in clinical interaction. Beyond these known neurodevelopmental and behavioral diagnoses, no additional detailed psychiatric history or documented psychiatric consultation could be recovered from the available record. She also had a seizure disorder beginning in adolescence and was receiving carbamazepine as her only documented antiseizure medication. The dose and the degree of recent seizure control were not available in the clinical information retained for this report. She had no history of prior abdominal surgery and no current or previous history of jaundice was reported.

On admission, the temperature was 38.1°C, heart rate was approximately 100-110 beats/min, blood pressure was 130/85 mmHg, respiratory rate was approximately 15 breaths/min, and oxygen saturation was greater than 95% on room air. During hospitalization, an additional febrile peak of 38.6°C was documented. The specific source of the prolonged fever was not established. Abdominal examination showed visible distension and increased bowel sounds. Palpation elicited pain predominantly in the epigastrium and mesogastrium, where a firm, poorly defined mass was appreciable. There were no clinical signs of peritoneal irritation.

Initial hematologic testing demonstrated leukocytosis with neutrophilia, bandemia, and mild thrombocytosis, without evidence of anemia. The available admission hematologic results are summarized in Table 1. Additional chemistry, inflammatory-marker, and microbiologic results were not available for the present report; consequently, no laboratory-based etiology for the fever is assigned.

**Table 1:** Admission Hematologic Findings *General adult reference intervals are provided for contextual interpretation because the original laboratory-specific reference intervals were not preserved; reference ranges may vary among laboratories.

Test	Result	Clinical Interpretation	Reference Range*
White blood cell count	15.6 × 10³/µL	Leukocytosis	4.5-11.0 × 10³/µL
Neutrophils	85%	Neutrophilia	40-70%
Bands	9%	Bandemia	0-5%
Platelets	456 × 10³/µL	Mild thrombocytosis	150-450 × 10³/µL
Hemoglobin	16.3 g/dL	Slightly above general reference interval; no anemia	12.0-16.0 g/dL (adult female)
Hematocrit	46.5%	Slightly above general reference interval	36-46% (adult female)

A plain abdominal radiograph demonstrated pronounced gastric distension. The gastric lumen contained a mottled radiolucent pattern with entrapped gas, described radiographically as a “bread-crumb” appearance, and the distended stomach displaced the transverse colon caudally (Figure 1). CT of the abdomen subsequently demonstrated marked gastric retention, with the stomach nearly completely occupied by a heterogeneous intraluminal solid mass containing areas of trapped gas and an air-fluid level (Figure 2). The dominant radiologic impression was a giant gastric bezoar/foreign-body conglomerate. No separate jejunal bezoar was prospectively identified on the preoperative CT.

**Figure 1:**
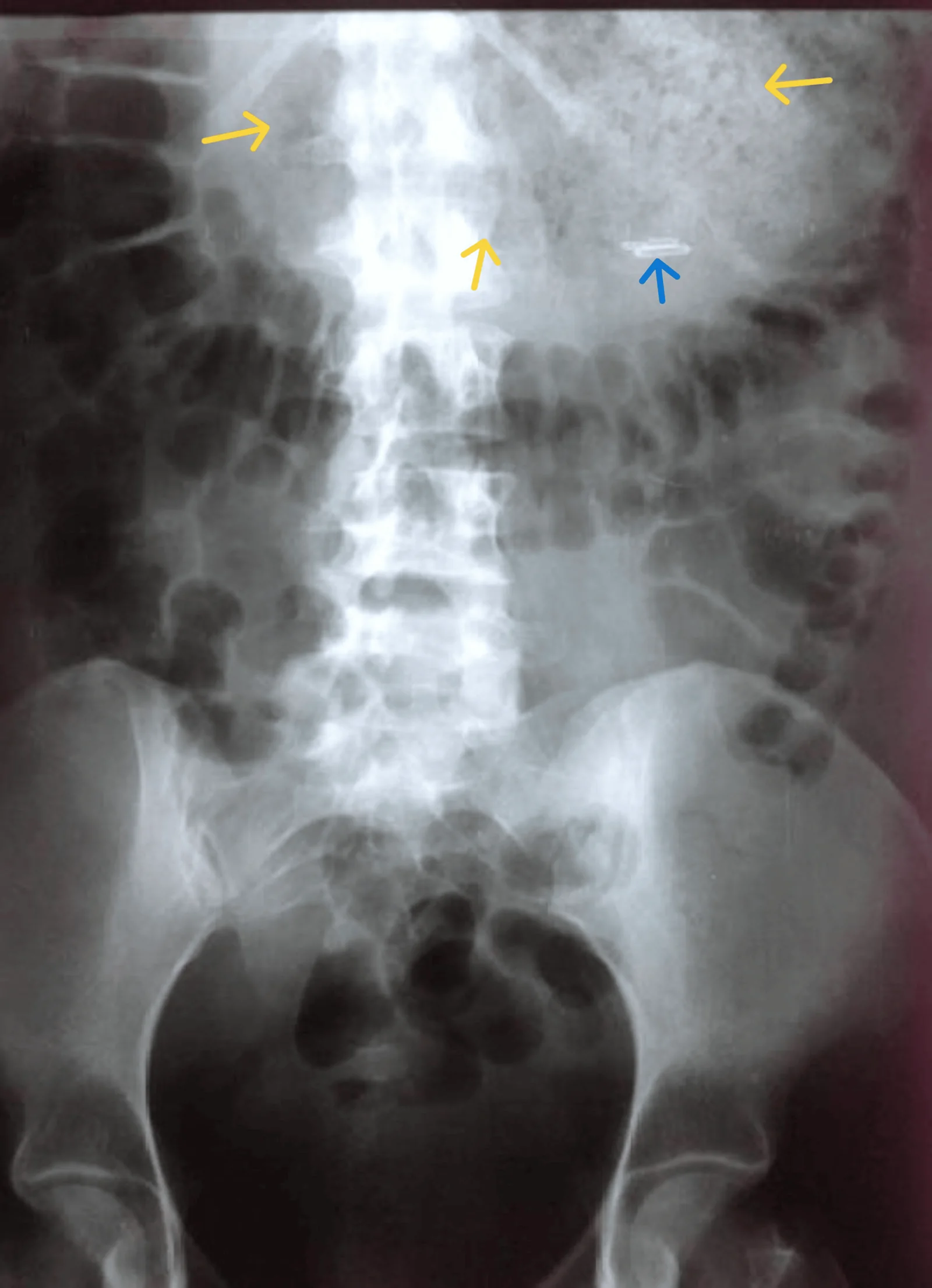
Plain Abdominal Radiograph Demonstrating Marked Gastric Distension, Intragastric Bezoar, and Metallic Staples Plain abdominal radiograph showing marked gastric distension with heterogeneous intragastric material consistent with a bezoar. The yellow arrows delineate the distended stomach, while the blue arrow indicates radiopaque metallic staples within the gastric contents, which were subsequently confirmed to be among the components of the surgically extracted mixed-material bezoar.

**Figure 2:**
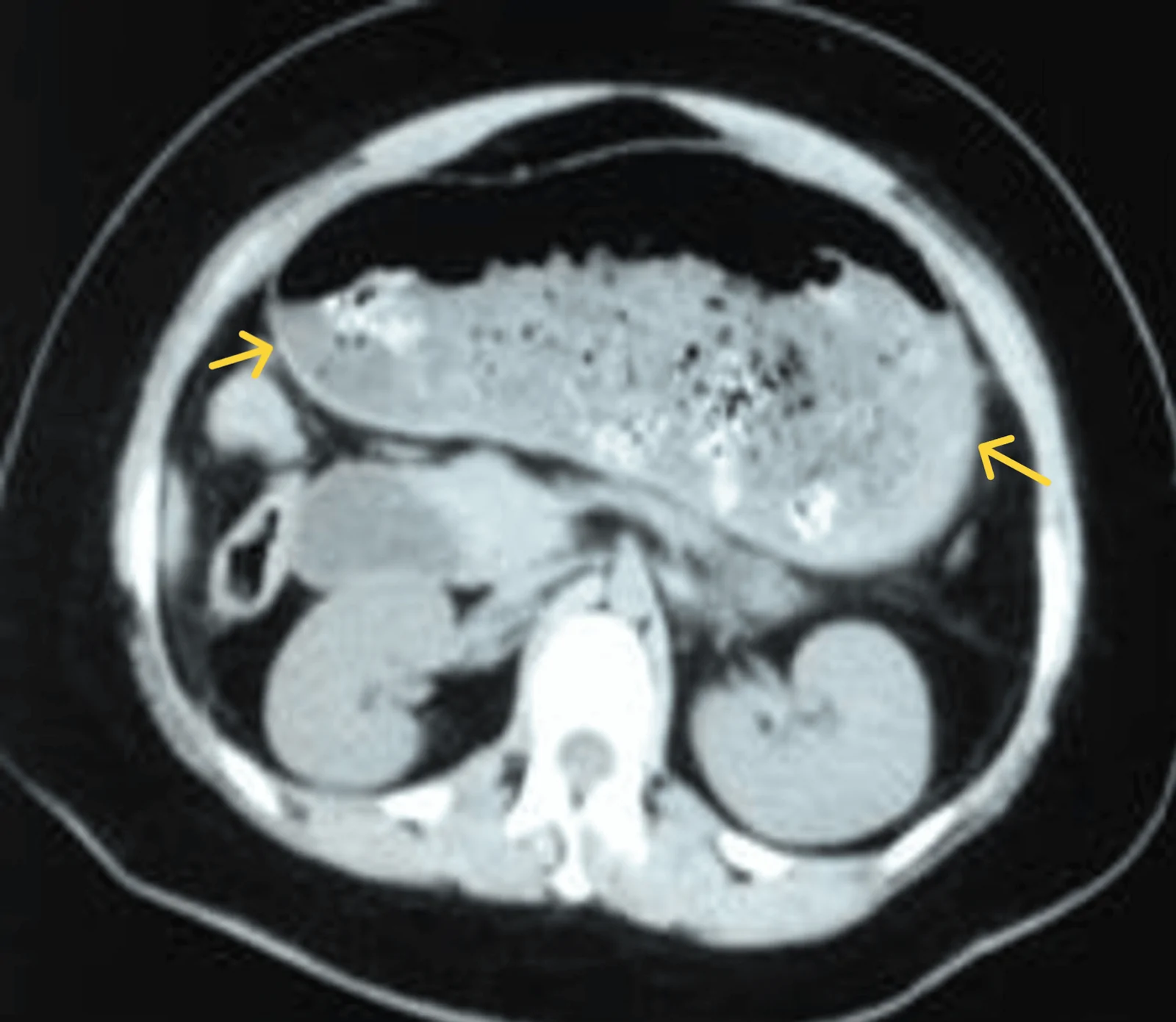
Abdominal Computed Tomography (CT) Showing a Giant Heterogeneous Gastric Bezoar Preoperative CT image demonstrating marked gastric dilatation with the stomach nearly completely occupied by a heterogeneous intraluminal mass containing entrapped gas and an air-fluid level, compatible with a giant gastric bezoar. The synchronous proximal jejunal bezoar was not prospectively identified on preoperative CT.

Upper gastrointestinal endoscopy confirmed a large foreign-body mass occupying the stomach. The examination was diagnostic only; no endoscopic fragmentation or extraction was attempted, and no endoscopic image could be recovered for publication. Given the extreme intragastric burden, with the stomach nearly filled by heterogeneous foreign material, together with rigid and nondegradable components suggested by the radiographic and endoscopic findings, complete endoscopic clearance was considered unlikely, while fragmentation raised concern for incomplete removal and distal migration. No reliable documentation was available regarding mucosal ulceration or active bleeding; these findings are therefore not attributed to the case. Operative management was selected for definitive removal.

Exploratory laparotomy revealed striking gastromegaly, with an intraoperative major gastric dimension of approximately 45 cm. The stomach was occupied by a large, soft-to-firm, heterogeneous foreign-body conglomerate. The proximal jejunum was also dilated and contained a second palpable intraluminal mass. This distal lesion was anatomically distinct from the gastric bezoar and represented an intraoperative finding rather than a preoperative radiologic diagnosis. No distal jejunal stenosis, fixed narrowing, or other obstructing lesion was identified intraoperatively.

A longitudinal anterior gastrotomy of approximately 15 cm was performed. The gastric contents were removed in fragments because of their volume and heterogeneous composition. The material extracted from the stomach had a measured weight of approximately 3,000 g. Attention was then directed to the proximal jejunum. A 5-cm enterotomy was created on the antimesenteric border over the second mass, allowing extraction of an additional approximately 500 g of similar mixed material. The gastric and jejunal masses were not physically connected. Both the gastrotomy and enterotomy were closed in two layers; the outer reinforcing layer was fashioned with a Lembert seromuscular technique. No additional operative detail regarding suture material or the configuration of the inner layer is included because it was not available in the retained case record.

The combined measured weight of the two bezoars was approximately 3.5 kg. Gross inspection demonstrated a remarkably heterogeneous composition: plastic bags, fragments of plastic bottles, plastic caps, candy wrappers, adhesive tape, hair, plant fibers, and metallic staples were identified among the extracted material (Figure 3). The bezoars were therefore considered mixed-material foreign-body bezoars rather than pure trichobezoars or phytobezoars. Because the material had been removed in multiple fragments, reliable maximum dimensions of either mass could not be obtained.

**Figure 3:**
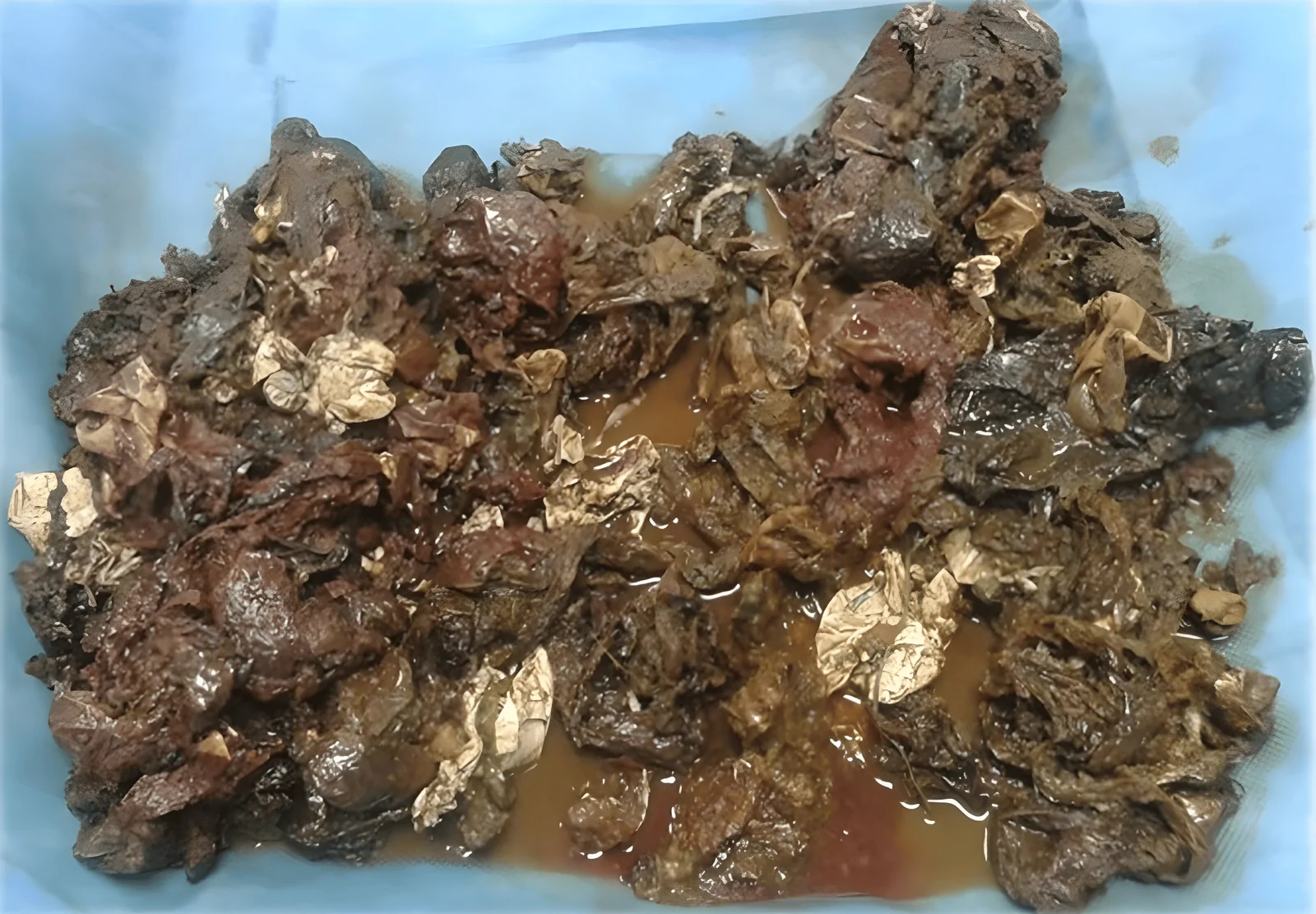
Extracted Gastric and Jejunal Mixed-Material Bezoars Gross appearance of the extracted gastric and jejunal bezoar material. The two anatomically separate masses had a combined measured weight of approximately 3,500 g and contained plastic bags, bottle fragments, plastic caps, candy wrappers, adhesive tape, hair, plant fibers, and metallic staples.

The postoperative course was uncomplicated. No routine contrast study was performed to evaluate the gastrotomy or jejunotomy closures. The absence of enteric leak was assessed clinically: there was no postoperative deterioration or abdominal finding suggestive of leakage, and no reintervention was required. There was no clinical evidence of surgical-site infection, intra-abdominal infection, or other postoperative complication. Oral intake was restarted on postoperative day 5 with liquids and was progressively advanced according to tolerance. By postoperative day 8, the patient was tolerating food and remained clinically stable; she was discharged in good condition. She was reassessed in the outpatient clinic approximately one week after discharge and again at one month. At both visits, recovery was favorable, oral intake was maintained, and no recurrent obstructive symptoms or surgical complications were documented. Whether formal psychiatric follow-up was subsequently performed could not be determined from the available records. The overall clinical course and key management milestones are summarized in Table 2.

**Table 2:** Clinical Timeline

Time Point	Clinical Event
~1 month before admission	Recurrent fever >38°C reported by family; colicky abdominal pain, distension, early satiety, constipation/reduced bowel movements, diaphoresis, and nonbilious alimentary vomiting.
Admission	T 38.1°C; HR 100-110 beats/min; BP 130/85 mmHg; RR ~15/min; SpO₂ >95%. Firm epigastric/mesogastric mass; no peritoneal signs. Hematologic evaluation showed leukocytosis with neutrophilia, bandemia, and mild thrombocytosis.
Initial imaging	Plain radiograph: marked gastromegaly and mottled intragastric gas/radiolucency. Computed tomography: stomach nearly filled by heterogeneous solid material; no separate jejunal bezoar prospectively identified.
Endoscopy	Large gastric foreign-body/bezoar mass visualized; endoscopy confirmed the diagnosis but did not provide definitive therapy.
Surgery	Exploratory laparotomy. ~45-cm gastromegaly. 15-cm anterior gastrotomy: 3,000-g gastric mixed-material bezoar removed. Separate proximal jejunal bezoar found intraoperatively; 5-cm antimesenteric enterotomy: 500-g second mass removed. Both closures two-layered with outer Lembert reinforcement.
Postoperative day 5	Oral liquids initiated and subsequently advanced according to tolerance.
Postoperative day 8	Tolerating food; no leak, infection, or other surgical complication; discharged in good condition.
1 week after discharge	Outpatient follow-up: favorable clinical recovery; no surgical complication documented.
1 month	Second follow-up: continued favorable recovery, maintained oral intake, no recurrent obstructive symptoms documented.

## Discussion

This case is best classified as a giant mixed-material gastric bezoar with a synchronous, physically separate jejunal bezoar. That terminology is important. Bezoars are often discussed according to a single dominant constituent, but patients with pica may ingest materials with markedly different physical properties, and the resulting conglomerate may not fit neatly within the classical phytobezoar/trichobezoar framework [1,3]. In the present patient, plastic predominated visually, but hair, plant fibers, adhesive material, and metal were also present. Contemporary case reports demonstrate that plastic and other nonfood materials can accumulate into large, mechanically significant masses and cause either gastric or small-bowel obstruction [4,8,9]. A 2024 report of a plastic bezoar in a 14-year-old girl with pica described material extending from the stomach toward the jejunum and ultimately requiring surgery [8], whereas another 2024 case documented plastic foreign objects in both the stomach and distal small bowel [9]. These cases underscore that nonfood bezoars should be conceptualized as a spectrum of foreign-body aggregation rather than as a single-material disease.

The association with pica and neurodevelopmental disorders is also central to the interpretation of this case. Pica is not synonymous with ASD, but epidemiologic data show a strong association, particularly when ASD coexists with intellectual disability [2]. In the study by Fields et al., pica was reported in 23.2% of children with ASD and in 28.1% of those with ASD plus intellectual disability, compared with 3.5% in population-based controls [2]. Although those data are pediatric and cannot be directly extrapolated as adult prevalence estimates, they demonstrate the magnitude of the association and support a low threshold for considering occult ingestion in patients with compatible developmental and behavioral histories. Schnitzler emphasized that pica may be associated with neurologic, developmental, psychiatric, nutritional, and environmental factors and that its medical consequences can be severe [3]. The current patient had a documented history of pica before presentation, which makes repeated ingestion the most coherent explanation for the extraordinary diversity and volume of foreign material.

Recent literature provides several clinically relevant parallels. Goh et al. reported small-bowel obstruction from a plastic bezoar in a young adult with autism and intellectual disability [4]. Palimaru et al. described a woman with autism, intellectual disability, and pica in whom a gastric bezoar altered drug absorption and contributed to serious toxicologic complications [5]. Gangadharan Nambiar et al. reported a paper-based xylobezoar in a patient with pica that was treated nonoperatively with prolonged enteral cola infusion [6]. Ba-Shammakh et al. described multiple bezoars containing hair and nonbiologic material in an autistic adolescent [7]. Together, these reports demonstrate that pica-associated bezoars vary widely in composition, anatomic distribution, and clinical behavior; therefore, management should be driven by the actual burden, location, material properties, and complications rather than by the diagnostic label alone.

A particularly notable feature of the present case is the presence of two independent bezoars. The gastric mass and jejunal mass were not connected by a tail or bridge of material. This distinguishes the presentation from classic Rapunzel syndrome. In Rapunzel syndrome, the defining morphology is a gastric trichobezoar with continuous extension through the pylorus into the duodenum or more distal small bowel. By contrast, this patient had a heterogeneous gastric bezoar and a second separate jejunal bezoar. The distinction is more than semantic: a continuous Rapunzel trichobezoar suggests extension of a single hair-based mass, whereas synchronous separate bezoars imply either repeated passage and impaction of independent material or multifocal aggregation. A 2024 report of a mixed gastrojejunal bezoar likewise illustrates that clinically important bezoar material can occur at more than one level of the gastrointestinal tract [10]. More recently, Febre-Alemañy et al. reported an adolescent with ASD who underwent open removal of gastric and distal duodenal/proximal jejunal trichobezoar material [11]. Their case and ours both reinforce the need to think beyond the stomach when the ingestion history or bezoar burden is substantial, although the composition and physical relationship of the masses differ. Selected contemporary case reports sharing major clinical features with the present case are summarized in Table 3.

**Table 3:** Selected Contemporary Reports Relevant to Mixed-Material, Multilevel, or Pica-Associated Bezoars ASD: autism spectrum disorder; CT: computed tomography

Report	Patient/Context	Location	Material	Management	Relevance to Current Case
Goh et al. (2022) [4]	Early-20s man; ASD + intellectual disability	Small bowel	Plastic glove bezoar	Laparotomy with bowel resection	CT findings were subtle; illustrates low-radiopacity of plastic and need for persistent clinical suspicion.
Ba-Shammakh et al. (2023) [7]	14-year-old girl; autism	Colon/rectum; multiple	Hair plus nonbiologic objects	Evacuation under anesthesia	Demonstrates multifocal mixed foreign material in a neurodevelopmental context.
Shrivastava et al. (2024) [8]	14-year-old girl; pica	Stomach with distal extension	Plastic	Surgical removal after endoscopic attempts	Large synthetic bezoars may be resistant to endoscopic clearance.
Sultan et al. (2024) [9]	58-year-old woman	Stomach and small bowel	Plastic	Exploratory laparotomy	Independent upper and distal foreign material supports attention to multiplicity.
Siqueira et al. (2024) [10]	17-year-old woman	Gastric + jejunal	Mixed gastrojejunal bezoar	Surgical treatment	Contemporary report of multilevel mixed bezoar disease.
Febre-Alemañy et al. (2026) [11]	17-year-old girl; ASD	Gastric + distal duodenal/proximal jejunal	Trichobezoar	Open gastrotomy + enterotomy	Useful comparator for ASD and multilevel disease; differs from the current mixed, physically separate masses.
Current case	21-year-old woman; ASD + mild intellectual disability + pica	Stomach + separate proximal jejunum	Mixed plastic, metal, hair, plant fibers, adhesive material	Laparotomy; gastrotomy + jejunotomy	3.5-kg measured total burden; jejunal lesion missed on preoperative CT; no physical continuity between masses.

The mechanism by which the second jejunal bezoar formed cannot be proven from the operative findings. Because the two masses were physically separate, several pathways are biologically plausible: repeated ingestion may have produced independent aggregates at different times; smaller gastric fragments may have traversed the pylorus and accumulated distally; or heterogeneous material may have separated according to size and deformability during transit. The case does not permit discrimination among these mechanisms, and no single explanation should be presented as established. What can be concluded is that the jejunal lesion was not simply the distal tail of the gastric mass. This distinction supports describing the process as synchronous multifocal bezoar disease rather than Rapunzel syndrome.

Preoperative imaging was informative but incomplete. Plain radiography in the current case demonstrated severe gastric distension and a mottled intragastric pattern caused by gas trapped within the mass. CT then defined the dominant gastric burden much more clearly. Heterogeneous intraluminal masses with entrapped gas are characteristic imaging findings in many bezoars, and CT is particularly valuable when obstruction, multiple lesions, ischemia, perforation, or an alternative diagnosis is being considered [1]. Nevertheless, the jejunal bezoar in this patient was not prospectively detected. This discrepancy is clinically instructive. The detectability of a bezoar depends on its composition, size, surrounding luminal contents, degree of obstruction, and the conspicuity of the transition point. Plastic and mixed low-attenuation material may be less visually obvious than a classic mottled phytobezoar, especially when a much larger gastric lesion dominates the study. Goh et al. similarly emphasized the diagnostic difficulty of plastic material on CT in a patient whose definitive diagnosis was established operatively [4].

The problem of multiplicity has direct operative implications. In a retrospective study of 188 patients with CT-confirmed bezoars, Muhtaroğlu et al. found that the jejunum was the most common localization and that enterotomy was frequently required for jejunal bezoars [12]. Although that cohort was dominated by a different risk profile and included many patients with previous gastrointestinal surgery, it demonstrates that small-bowel localization is not exceptional once bezoar disease is established. When a massive gastric bezoar is associated with a history of repeated nonfood ingestion, clinicians should therefore maintain suspicion for additional distal material even if only one dominant lesion is evident preoperatively. This does not mean that every patient requires laparotomy or that imaging is intrinsically unreliable. Rather, if surgery is undertaken, systematic assessment for additional obstructing intraluminal masses is a rational component of operative judgment, particularly when there is unexplained distal dilation or a high-risk ingestion history.

Upper endoscopy has two important roles: confirmation of the diagnosis and, in selected patients, definitive treatment. Contemporary endoscopic reports demonstrate that increasingly large bezoars can sometimes be fragmented and removed without surgery. Choe et al. described successful endoscopic treatment strategies across three gastric bezoar cases [13]. Hu et al. reported a guidewire-based tangential sawing technique for giant gastric bezoars, reflecting the evolution of advanced endoscopic fragmentation [14]. Matsuura et al. achieved complete endoscopic removal of a large trichobezoar using a prolonged, technically demanding multimodal endoscopic approach [15]. These experiences are important because “large” or even “giant” does not automatically equal “surgical” in every center.

Chemical dissolution is also useful for selected gastric bezoars, especially phytobezoars. Delgado Galan and Rabago reviewed the evolving role of cola-based dissolution and highlighted both its utility and the heterogeneity of published evidence [16]. Gangadharan Nambiar et al. demonstrated successful dissolution of a xylobezoar using continuous enteral cola infusion [6]. However, these approaches are strongly dependent on composition. Plastic bottle fragments, caps, adhesive tape, metallic staples, and large quantities of hair are not expected to undergo meaningful chemical dissolution. Similarly, fragmentation of a 3-kg heterogeneous gastric foreign-body conglomerate could create a risk of distal migration, mucosal injury, incomplete clearance, or persistent small-bowel obstruction.

Management of bezoars can be considered along a spectrum of conservative/chemical, endoscopic, and surgical approaches. Conservative dissolution is most applicable to selected gastric phytobezoars, while endoscopic fragmentation or retrieval is appropriate when the material can be safely disrupted and completely cleared [13-16]. Surgical treatment may be minimally invasive or open. Laparoscopy is a reasonable option in selected patients with a localized bezoar that can be manipulated and extracted through a limited gastrotomy or enterotomy, but very bulky lesions may be technically difficult and may require conversion to laparotomy [1]. Direct comparative outcome data between laparoscopic and open surgery are limited because the literature is dominated by case reports and small series; therefore, approach selection is individualized according to bezoar composition, size and total burden, location, bowel distension or compromise, patient factors, and local expertise [1,17,18]. In the present case, a formal laparoscopic plan was not documented. The marked gastromegaly, 3,000-g heterogeneous gastric mass with rigid synthetic and metallic components, and need for reliable extraction of a very large nondegradable burden favored an open approach; the separate 500-g jejunal bezoar was then identified and treated during exploration. These case-specific factors, rather than size alone, explain the chosen operative strategy.

The mixed plastic-metal component of this case is also unusual. Most bezoar literature remains dominated by phytobezoars and trichobezoars. Recent case reports, however, document clinically consequential metallic and plastic conglomerates. Flores Garcia et al. described metal-bezoar-induced gastrointestinal obstruction requiring surgical management [19], while the plastic bezoar reports by Shrivastava et al. and Sultan et al. illustrate the mechanical persistence of synthetic material [8,9]. Our patient's combination of bottle fragments, caps, bags, wrappers, tape, staples, hair, and vegetable fibers creates a bezoar phenotype for which standard dissolution algorithms developed for phytobezoars are poorly applicable. Describing the actual composition is therefore essential because “bezoar” alone does not predict endoscopic fragility or chemical solubility.

The measured weight of 3.5 kg adds to the clinical significance of the case but should be interpreted carefully. Published reports use inconsistent definitions of “giant,” and bezoars are variously reported by weight, maximum dimension, percent gastric occupancy, or extension into the bowel. For that reason, the present report emphasizes the measured weight rather than claiming a specific record. A recently published case of a giant gastric trichobezoar in a 27-year-old woman required laparotomy and anterior gastrotomy and illustrates that exceptionally large gastric masses remain a contemporary surgical problem [20]. The current case differs because the 3.5-kg total burden was composed of heterogeneous nonfood material distributed in two separate anatomic locations rather than a single hair-based Rapunzel mass.

The patient's fever and inflammatory hematologic profile deserve cautious interpretation. She presented with prolonged fever, leukocytosis, neutrophilia, bandemia, and thrombocytosis. However, no definitive infectious source is available in the retained clinical record, and no postoperative infection occurred. It would therefore be inappropriate to label the bezoar itself as infected or to infer occult perforation or bacterial translocation without evidence. Fever may coexist with dehydration, inflammation, aspiration, infection outside the gastrointestinal tract, or other processes, but the present case cannot distinguish among them. Preserving that uncertainty is preferable to assigning a causal mechanism that was not documented.

The postoperative course was favorable despite the size of the operation. Oral intake resumed on postoperative day 5, discharge occurred on postoperative day 8, and follow-up at one week after discharge and at one month documented continued clinical recovery without leak, infection, recurrent obstruction, or other surgical complications. The absence of early recurrence is reassuring but does not eliminate long-term risk. Mechanical treatment removes the consequence of pica, not the behavioral tendency itself. Recurrence prevention should therefore include assessment of the drivers of pica, review of nutritional or micronutrient contributors when clinically appropriate, behavioral and psychiatric interventions adapted to the patient's cognitive profile, family/caregiver education, and reduction of access to repeatedly ingested hazardous objects [2,3,7]. For patients with ASD and intellectual disability, prevention strategies may need to be individualized and caregiver-mediated.

Several limitations should be acknowledged. First, the exact dimensions of the gastric and jejunal bezoars were not recorded because the material was removed in fragments; measured weight is therefore the most reliable quantitative descriptor. Second, the retained endoscopy report did not provide sufficient detail to characterize the gastric mucosa, and no endoscopic image could be recovered for publication. Third, the available intraoperative photograph obtained during bezoar extraction was severely pixelated and unsuitable for publication, and no diagnostically adequate intraoperative image of the palpable jejunal mass was available; therefore, these images were not included. Fourth, the etiology of the prolonged fever was not established. Fifth, biochemical data beyond the available complete blood count were not recoverable for this report. Finally, documented follow-up extends to one month, and whether structured psychiatric follow-up occurred after discharge could not be determined from the available records; consequently, the report can describe early surgical recovery but not long-term recurrence prevention or psychiatric management. These limitations do not alter the central observations: an extreme mixed-material gastric burden, a separate synchronous jejunal bezoar missed on preoperative CT, successful open removal, and uncomplicated short-term recovery.

## Conclusions

Giant bezoars in patients with pica may contain heterogeneous synthetic, metallic, organic, and hair components that make standard dissolution or routine endoscopic extraction impractical. In this patient, a 3,000-g gastric mixed-material bezoar coexisted with a physically separate 500-g proximal jejunal bezoar that was not identified on preoperative CT. The absence of continuity between the two masses distinguishes the presentation from classic Rapunzel syndrome. Definitive open removal through gastrotomy and jejunotomy produced an uncomplicated recovery.

When a dominant gastric bezoar is encountered in a patient with repetitive nonfood ingestion, clinicians should consider the possibility of additional distal bezoars, particularly if obstructive symptoms or bowel dilation are present. Cross-sectional imaging and endoscopy remain central to diagnosis and treatment planning, but operative findings may reveal clinically important synchronous disease. Long-term management should extend beyond removal of the bezoar and address pica through individualized behavioral, psychiatric, nutritional, and caregiver-based strategies intended to reduce recurrence.
